# Space Matters: Estimating Species Diversity in the Fossil Record

**DOI:** 10.1371/journal.pbio.0030286

**Published:** 2005-07-19

To get a sense of the challenges in measuring biodiversity, consider this: estimates for the number of living species on earth range from 3.5 million to over 30 million. Only 1.9 million species have been classified and described. (Of these, 15,589 currently face extinction.) Now imagine trying to calculate patterns of biodiversity through the paleontological record. One tool ecologists rely on to identify patterns of biological diversity is a long-established rule of thumb called the species–area effect: the tendency for species number, or richness, to increase in a regular way with area.

Paleontologists typically have been unable to apply the species–area rule in estimating paleodiversity and instead use species counts for a given time interval to calculate historical biodiversity, with the assumption that other sampling considerations override the species–area effect. In a new study, Anthony Barnosky, Marc Carrasco, and Edward Davis test this assumption and discover that the golden rule of ecology holds for the rock record as well. Just as geographic sampling influences diversity counts in the modern landscape, the species–area effect influences counts in the fossil record.

To get a true picture of geographic conditions, Barnosky et al. used mapping and imaging systems that generate direct measures of the geography for a given set of fossil species. To get a sense of diversity across time and space, the authors used a recently completed archival database (which they also built) that integrates the geographic data with fossil datasets, called the Miocene Mammal Mapping Project (MIOMAP). MIOMAP includes all western North American mammals from 5–30 million years ago—3,100 localities and 14,000 occurrences of species in all.

The authors first tested the fossil data for species–area effects with species from a time period with robust geographic data (the Early Barstovian, about 14.8–15.9 million years ago). They plotted species richness against geographic area, using what's known as nested sets of fauna (species that occur in a smaller area represent a subset of species in a larger area) by starting with one biogeographic region and successively adding species from others. They also used unnested sets of fauna to plot species richness within a given time period for nine different geographic regions against the geographic sampling area. After correcting for possible biases in sample size that might influence the number of species, Barnosky et al. found a strong species–area effect in both analyses.

These results, they argue, suggest that many fluctuations in diversity seen in fossil analyses actually arise from the species–area effect. Given the lack of uniform geographic sampling in paleontological data, the impact of this effect may be significant—and likely applies to other taxa as well. Once the effect is factored in, one might expect significant adjustments in accepted patterns of global and regional paleodiversity. And because an important metric for understanding current extinctions relies on descriptions of past extinction events, controlling for a paleodiversity–area effect may provide a better frame of reference for understanding the current biodiversity crisis. Estimates of paleodiversity also have important evolutionary implications for understanding how and when new species emerge. Thanks to the innovative text-mining tools and approach presented here, future studies can more easily correct for area effects and explore these issues. And given the parallels between species–area relationships in paleontology and ecology, collaborations across disciplines may offer valuable insights into ecological dynamics through time. [Fig pbio-0030286-g001]

**Figure pbio-0030286-g001:**
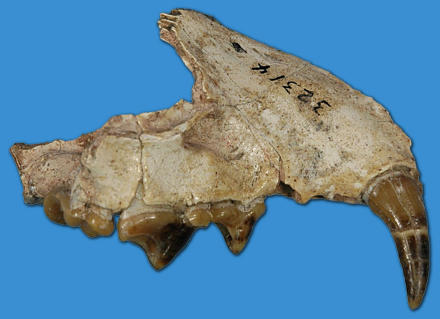
A new, comprehensive database compiles mammalian fossils including this upper jaw of the Sthenicitis campestris, a weasel from about 12 million years ago (Photo: Alan B. Shabel)

